# The Role of Uncontrollable Trauma in the Development of PTSD and Alcohol Addiction

**Published:** 1999

**Authors:** Joseph Volpicelli, Geetha Balaraman, Julie Hahn, Heather Wallace, Donald Bux

**Affiliations:** Joseph Volpicelli, M.D., Ph.D., is an associate professor and senior scientist, Geetha Balaraman, is a research associate, Julie Hahn, is a research associate, and Heather Wallace, M.A., is a research specialist, all at the Treatment Research Center at the University of Pennsylvania, Philadelphia, Pennsylvania. Donald Bux, Ph.D., is an instructor at the Center for the Treatment and Study of Anxiety at the University of Pennsylvania, Philadelphia, Pennsylvania

**Keywords:** trauma, learned helplessness, endorphins, post traumatic stress disorder, AOD (alcohol or other drug) use, psychological stress, physiological stress, adrenocorticotropic hormone, corticotropin RH, gender differences, treatment, literature review

## Abstract

After a traumatic event, people often report using alcohol to relieve their symptoms of anxiety, irritability, and depression. Alcohol may relieve these symptoms because drinking compensates for deficiencies in endorphin activity following a traumatic experience. Within minutes of exposure to a traumatic event there is an increase in the level of endorphins in the brain. During the time of the trauma, endorphin levels remain elevated and help numb the emotional and physical pain of the trauma. However, after the trauma is over, endorphin levels gradually decrease and this may lead to a period of endorphin withdrawal that can last from hours to days. This period of endorphin withdrawal may produce emotional distress and contribute to other symptoms of posttraumatic stress disorder (PTSD). Because alcohol use increases endorphin activity, drinking following trauma may be used to compensate this endorphin withdrawal and thus avoid the associated emotional distress. This model has important implications for the treatment of PTSD and alcoholism.

Margaret was raised in a chaotic environment, experiencing extensive physical abuse first by her alcoholic parents, then later in an abusive relationship. During one particularly disturbing event, she recalled being severely beaten, then locked in a closet, bleeding, for several hours. She also recalled sexual abuse by her intoxicated father from the ages of 8 to 14. When Margaret was 16 she was involuntarily hospitalized following a suicide attempt, and subsequently became involved in a sexual relationship with a male patient who forced her to participate in group, sadomasochistic sex several times during a 6-month period. Following this experience, Margaret began abusing a variety of substances, primarily alcohol. When she presented for treatment at age 38, she had undergone at least 10 prior treatment attempts for alcohol dependence. She reported drinking up to a case of beer daily, which she said she used primarily to help her sleep and to suppress nightmares of the sexual abuse, and also in response to the trauma reminders she frequently experienced in daily life. When abstinent from alcohol, Margaret reported extremely vivid and disturbing nightmares, profound agitation and jumpiness, and acute reactivity to a variety of environmental cues that reminded her of her traumatic experiences.

Unfortunately, this example is far too common, as people like Margaret, after an experience of sexual or physical victimization, turn to alcohol to relieve symptoms of anxiety, irritability, and depression. In this paper we present a new model to help explain how trauma’s effects on psychological distress may influence alcohol consumption.

The experience of psychological trauma (experiencing or witnessing an event involving actual or threatened death or serious injury of self or others [APA 1994]) does not necessarily lead to long-term emotional distress or alcohol abuse. Rather, the likelihood of experiencing adverse consequences is related to the victim’s ability to cope with the trauma. Consider the following two hypothetical examples. Barbara and Jan both attend a seminar on crime prevention. On her way home, Barbara encounters a man who points a gun to her head and demands her money. Caught off guard, Barbara freezes in terror, forgetting everything she has just learned in the class about how to protect herself; the assailant takes her pocketbook and runs off with $50 and all of Barbara’s credit cards. Although Barbara avoided physical harm, she was left with the feeling that she had no control over the outcome of the incident (i.e., she experienced *uncontrollable* trauma) and, as a result, experienced feelings of terror and helplessness. Compare Barbara’s situation to Jan’s. On her way home from the same crime prevention class, Jan encounters another man who points a gun at her head and demands her money. Jan is also afraid but manages to keep her wits, and recalling one of the strategies she just learned in the class, she throws her pocketbook past the gunman down the street. The assailant runs after the pocketbook, and Jan runs in the opposite direction. In comparison to Barbara, Jan experienced *controllable* trauma, because she took direct action that influenced the outcome of the incident and provided the means for her own escape. Both women experienced similar emotions and tangible loss as a result of the incident, and both escaped unharmed, but Barbara felt a pervasive sense of helplessness after the crime and felt that her self-defense class had been in vain, whereas Jan felt a sense of control over the trauma because of her swift action that enabled her to escape. As a result, Barbara experienced more severe post-traumatic symptoms than did Jan.

To understand how trauma can lead to emotional distress and affect alcohol consumption, it is important to understand the biochemical changes that occur during and after an experience of uncontrollable trauma. During uncontrollable trauma, an increase in endogenous opioids (endorphins) helps to numb the pain of the trauma. Following the trauma, however, a rebound endorphin withdrawal can contribute to the symptoms of emotional distress observed after a traumatic event as well as an increased desire to drink alcohol. The endorphin compensation hypothesis assumes that people use alcohol following a traumatic experience in an attempt to relieve the endorphin deficiency. According to this hypothesis, this use of alcohol creates a vicious cycle in which more alcohol is needed to prevent subsequent endorphin withdrawal symptoms. Chronic exposure to this addictive cycle can lead to alcohol addiction. Special populations, such as women, may be at particular risk for trauma-induced, co-occurring alcoholism and psychopathology. This model has important implications for the treatment of trauma-induced psychological distress and alcohol addiction.

## Posttraumatic Stress Disorder

People who have been exposed to an extremely traumatic event, such as witnessing a death, having one’s life threatened, or enduring serious injury, may develop a set of symptoms known as posttraumatic stress disorder (PTSD). PTSD is diagnosed in people who are exposed to a potentially life-threatening trauma, during which they experience an acute sense of intense fear, horror, or helplessness. People afflicted with PTSD experience symptoms from the following three clusters:

- A reexperience of the event through nightmares, flashbacks, and so forth.
- Active and passive avoidance of the event. Active avoidance occurs when sufferers make a point of avoiding situations, places, and people who remind them of the trauma. Passive avoidance, or numbing of responsiveness, is the body’s way of avoiding the event through feelings of detachment or estrangement from the external world.
- Generalized hyperarousal, or increased arousal. Sufferers may experience difficulty falling asleep or staying asleep, outbursts of anger, and exaggerated startle responses (APA 1994).

Margaret experienced symptoms from all three clusters. For example, she reexperienced her sexual abuse through frequent nightmares and intrusive, distressing thoughts and images of the event whenever she encountered men who physically resembled her father or when she was in closed spaces, such as closets or basements. Emotionally, she alternated between feeling apathetic, numb, and alienated from others and feeling “on edge,” hypervigilant, and anxious. She avoided numerous situations reminiscent of her earlier experiences, including her childhood home and movies and news items involving child abuse. She also avoided discussing her abuse history with others and attempted to suppress her own memories of what happened. She felt unable to control many of these PTSD symptoms except by drinking alcohol, but even alcohol provided only temporary relief.

## Trauma and Learned Helplessness

Research in the past quarter century has shown that experiencing trauma does not necessarily lead to psychopathology. It is not uncommon for people to experience a traumatic event. As much as 70 percent of the U.S. population has experienced at least one trauma, such as a traffic accident, assault, or an incident of physical or sexual abuse. Many people are able to cope with their traumatic experiences and do not suffer from prolonged consequences. For about 8 percent of the population, however, the consequences of experiencing trauma do not abate and may indeed get worse with time (Breslau et al. 1991; Kessler et al. 1995). The degree to which a person or animal can control a traumatic event is an important factor in understanding the impact of the event (Seligman 1975). In fact, an event can have very different effects depending on the victim’s ability to cope with the event. Children who are victimized have very little control over the traumatic event and may experience severe emotional distress as a result.

After experiencing uncontrollable traumatic events, animals and humans show physiological, behavioral, and emotional symptoms of distress. For example, rats that have been exposed to shocks that they cannot control often become strikingly passive when later placed in new traumatic situations. They appear numb to the new trauma as if they have “given up.” Alternatively, they also become especially fearful of environments where they experience similar traumas and will try to avoid such situations. Seligman and colleagues termed this behavior “ learned helplessness” (Maier and Seligman 1976).

In addition, animals exposed to uncontrollable footshocks, unlike animals exposed to electric footshocks that they can escape, show depletion in catecholamines and elevations in stress hormones. These biochemical changes are associated with organic diseases such as gastric ulceration (Weiss et al. 1970), immunosuppression (Laudenslager et al. 1983), and even decreased resistance to the injection of tumor cells (Visintainer et al. 1982). For example, in one study rats were given injections of cancer cells following experience with shocks that were either uncontrollable, controllable, or nonexistent. The rats that received the uncontrollable shock (i.e., the learned helplessness rats) were less able to resist the cancer cells, and less than one-third survived. In contrast, some rats experienced the same intensity electric foot-shock but could turn off the shock by pressing a lever. Two-thirds of these rats survived. Other rats were placed in the shock compartments but experienced no shocks. A little more than half of these rats survived (Visintainer et al. 1982).

## Learned Helplessness and PTSD

The grouping of symptoms that follow experience with uncontrollable trauma is called “ learned helplessness effects” (Seligman 1975). As described above, animals that experience uncontrollable trauma learn that their responses are of no consequence, leaving them helpless to cope with a traumatic situation.

Although originally proposed as an animal model of depression, learned helplessness has many similarities with PTSD. Both PTSD and learned helplessness develop following exposure to negative stressors or uncontrollable events. In addition, learned helpless animals and patients with PTSD suffer from a variety of similar behavioral symptoms, such as increased generalized arousal (marked by exaggerated startle response or hypervigilance). For example, someone with PTSD may easily jump when he or she hears a car backfire, responding as if a gun had been fired. Similarly, after experiencing uncontrollable trauma, rats will be fearful in an open field and hesitant to venture out and explore the environment. Both conditions are associated with anticipatory fear prompted by situations that resemble the traumatic event. If reexposed to the traumatic event, both animals and humans more easily “give up” and appear strikingly passive. The biochemical changes observed in animals following uncontrollable trauma parallel those changes sometimes seen in humans following uncontrollable traumatic events. Stress hormone levels are often elevated between episodes of trauma. Also, as is the case with animals exposed to uncontrollable stress, patients with chronic PTSD have high rates of medical problems, such as autoimmune diseases (van der Kolk 1997).

Another similarity between learned helplessness as seen in animal models and PTSD is the co-occurrence of excessive alcohol consumption. In an experiment in which some rats were exposed to shocks they could escape from and others were exposed to shocks that were inescapable, rats that were presented with inescapable shocks increased alcohol preference compared with rats that received escapable shocks (Volpicelli 1987; Volpicelli and Ulm 1990). The rats’ alcohol consumption did not increase on the days that they experienced the shocks, however, but did increase 1 day later.

The behavioral and physiological similarities between learned helplessness in animals and patients with PTSD suggest that learned helplessness is a good model to understand PTSD (see Foa et al. 1992 for a review).

## Poststress Alcohol Consumption

In both animals and humans, traumatic events and increased alcohol consumption are clearly related; but alcohol use typically increases following the trauma, rather than during the trauma. Much of the confusing literature on stress and alcohol use is understood better when one assesses alcohol use in relationship to when the trauma occurred. For example, in a study with rats we found very modest increases in alcohol consumption on days when shocks were administered but dramatic increases in alcohol preference on subsequent days (Volpicelli et al. 1990). We termed this the “ happy hour effect” and have noted that even among social drinkers, alcohol consumption increases following, but not during, exposure to stress. These results were the opposite of what we expected based on a tension-reduction theory of alcohol use. If one uses alcohol solely to reduce anxiety, alcohol consumption should increase during times of stress rather than after the stress.

Research in humans has also identified a strong association between PTSD and alcoholism. For example, in a sample of Vietnam combat veterans with PTSD, more than half subsequently showed signs of alcohol addiction (Bremner et al. 1996). Similarly, women exposed to childhood rape often report turning to alcohol to reduce symptoms of PTSD (Epstein et al. 1998). In addition, investigators found that 40 percent of inpatients receiving treatment for substance abuse also met criteria for PTSD (Dansky et al. 1997).

## Biology of the Stress Response

**Figure 1:**
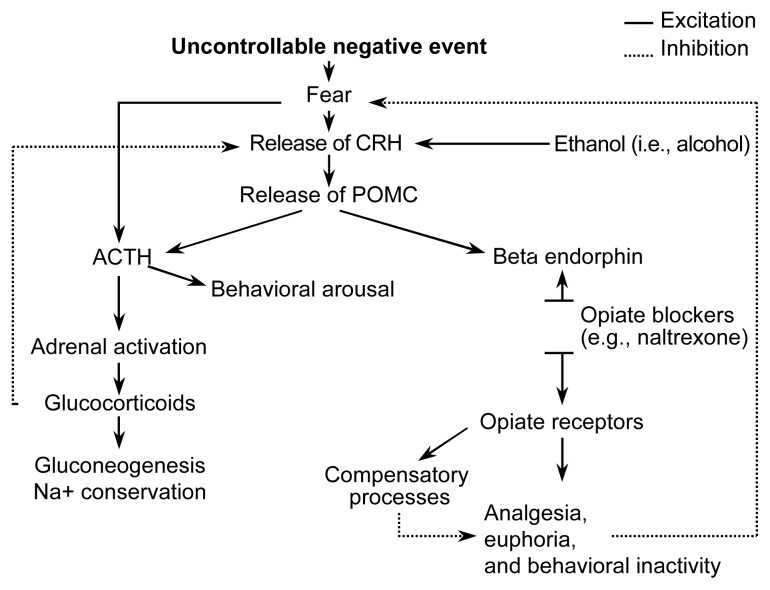
Biochemical responses to stress. Exposure to an uncontrollable negative event elicits the familiar “fight-or-flight” response. Fear prompts the release of corticotropin-releasing hormone (CRH). In turn, CRH stimulates the release of proopiomelanocortin (POMC), a hormone that is divided into several components. These components include adrenocorticotropic hormone (ACTH), which increases arousal and produces the fight-or-flight response, and beta-endorphin, which has a numbing effect and thereby reduces both emotional and physical pain.

Why does alcohol preference increase after stress, rather than during stress? The biology of the stress response gives us a clue. As shown in figure 1, exposure to uncontrollable stress elicits the familiar “fight or flight” response. Fear prompts the release of corticotropin-releasing hormone (CRH), which in turn stimulates the release of proopiomelanocortin (POMC), a large molecule that is broken up into several parts including adrenocorticotropic hormone (ACTH), which is responsible for the familiar “fight or flight” response, and beta-endorphin, which may have the survival advantage of numbing pain if the organism is attacked. The ACTH limb of the stress response increases arousal. The beta-endorphin limb causes a reduction in emotional and physical pain.

## The Role of Endorphins in PTSD and Alcohol Drinking

The model presented above suggests that uncontrollable trauma in humans and other mammals should lead to a release of endorphins and increased numbing. Research has shown that when animals are presented with inescapable shocks, their pain response decreases (Jackson et al. 1979). The reduced pain response is attributable to an increased release of endogenous opioids (Hyson et al. 1982; Williams et al. 1984). When rats were given naltrexone, which blocks the effects of endorphins, the numbing effects of uncontrollable trauma were also blocked. Furthermore, after uncontrollable trauma naltrexone produced an opiatelike withdrawal reaction suggesting a withdrawal from one’s endorphins (Hyson et al. 1982). Although exposure to controllable trauma, produces a modest analgesia, it is brief and there is no endorphin withdrawal following the traumatic experience (Hyson et al. 1982). This shows that endorphin activity increases in response to uncontrollable trauma, but not in response to the same traumatic event if it can be controlled, a finding with important implications for understanding endorphin activity associated with PTSD.

Human studies have also shown that traumatic events can increase endorphin activity. For example, patients with PTSD will experience numbness or analgesia when simply exposed to reminders of the trauma (Pitman et al. 1990). We know the analgesia is attributable to a release of endorphins because drugs that block endorphins (opioid blockers) also block the analgesia in PTSD patients. In one study, Vietnam veterans with PTSD were shown a videotape of combat and asked to rate the pain intensity of a hot stimulus. After viewing the videotape the hot stimulus was less painful (i.e., the trauma reminder produced analgesia). However, when the opioid receptors were blocked with naloxone, an injectable opioid receptor blocker, there was no analgesia (van der Kolk et al. 1989). The naloxone blocked the analgesia produced by the trauma reminder; and, with their opioid receptors blocked, patients with PTSD felt the pain as severely as did people who did not have PTSD. This finding shows that trauma reminders in PTSD patients activate the endorphin system.

## Endorphin Compensation Hypothesis

Chronic stimulation of the stress response leads to two compensatory responses. First, ACTH tends to inhibit the further release of CRH. Second, chronic stimulation of opioid receptors leads to an increase in an opposing system that has anti-opioidlike effects. Over time, the opposing system gets stronger and this leads to a lessening or habituation of the endorphin system. But when the trauma is over, the net result is a deficit in endorphin functioning and a resultant endorphin withdrawal.

Like traumatic events, alcohol use can increase endorphin activity. In this way, drinking can compensate for the endorphin withdrawal that follows a traumatic experience. The endorphin compensation hypothesis (ECH) suggests that when people drink alcohol after traumatic events, the alcohol makes up for the lack of endorphin activity (Volpicelli 1987). According to this hypothesis, rats exposed to uncontrollable shocks should consume more alcohol than rats exposed to controllable shocks to compensate for the lack of endorphin activity that occurs after experiencing uncontrollable shocks. This explains why alcohol consumption would increase after the trauma, not before (in anticipation) or during the trauma, as predicted by the tension-reduction hypothesis.

Some people who either experience several traumatic events or continually reexperience the same event, as people with chronic PTSD do, will drink to reproduce the numbing effects experienced with increased levels of endorphins. The constant reexperiencing of the PTSD symptoms causes an initial increase in endorphin activity followed by a rebound withdrawal. During the rebound withdrawal, craving for alcohol should increase. One study conducted with Vietnam combat veterans with chronic PTSD showed that their alcohol use generally began after the onset of PTSD symptoms. For many of the patients, alcohol consumption continued to increase as their symptoms of PTSD increased (Bremner et al. 1996).

## Women’s Increased Risk for Trauma-Induced Emotional Distress and Alcoholism

The association between PTSD and alcoholism is particularly strong for women. In adults, the rates for co-morbid PTSD and substance use disorders are two to three times higher for females than males, with 30 to 57 percent of all female substance abusers meeting the criteria for PTSD (Najavits et al. 1997). Women’s increased risk for co-morbid PTSD and substance dependence is related to their higher incidence of childhood physical and sexual abuse. For example, in a group of adolescents, a history of sexual abuse increased the risk of problem drinking to 20 times the normal rates of alcohol abuse for both sexes. However, females were much more likely to have been sexually abused than males and consequently the symptoms of PTSD were more common for female than male alcohol abusers (Clark et al. 1997).

These early experiences of physical or sexual abuse can have a life-long effect. Early experience with trauma (e.g., a history of childhood sexual or physical abuse) also heightens a person’s susceptibility to severe PTSD symptoms as an adult. For example, victims of childhood physical and sexual abuse are at higher risk for developing PTSD symptoms following traumatic events in adulthood (Breslau et al. 1999).

## Implications for Treatment of PTSD and Alcoholism

**Figure 2:**
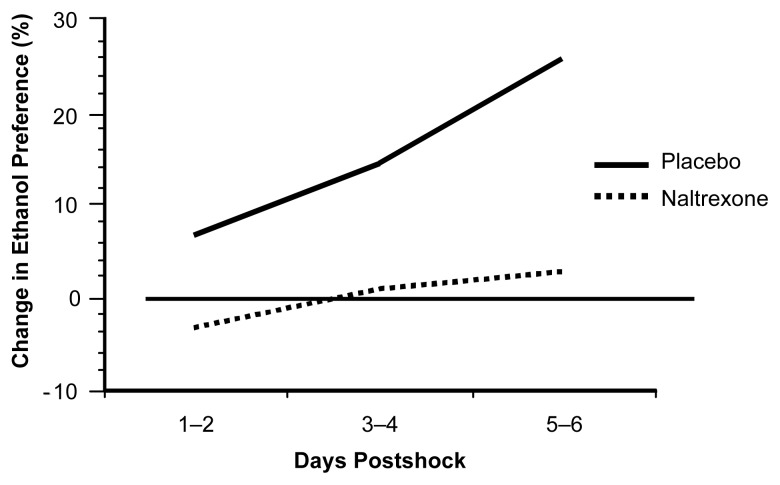
Naltrexone and postshock alcohol preference. The opioid blocker naltrexone blocked the poststress increase in alcohol consumption. SOURCE: Volpicelli et al. 1986.

The treatment of PTSD patients with alcohol dependence involves simultaneously addressing both disorders, because they seem to be intertwined. In therapy, patients learn to cope with their previous traumas and to handle situations that may remind them of the event. In this way, the patients learn how to better control or avoid such situations. Because research shows that both alcohol use and trauma increase endorphin activity, opioid receptor blockers may be a useful part of treatment for PTSD. We speculate that as trauma-related memories brought up during therapy may cause a release of endorphins and subsequent emotional numbing, this may interfere with the patient’s ability to engage in therapy fully. We also speculate that as endorphin levels decrease after the therapy session ends, endorphin withdrawal may lead to increased alcohol craving. Although alcohol use may temporarily relieve PTSD symptoms, alcohol withdrawal intensifies such symptoms. To avoid the increase in PTSD symptoms following a bout of drinking, the patient is caught in a vicious cycle in which he or she must continue to drink to avoid the unpleasant reaction following an alcoholic binge. An opioid antagonist such as a naltrexone would block the endorphin response and reduce the desire for alcohol. In an animal study (Volpicelli et al. 1986), we have shown that the opioid blocker naltrexone can prevent increased alcohol consumption following trauma. Rats will typically increase their alcohol consumption after several days of 1-hour sessions of brief electric footshocks. However, as shown in figure 2, the use of naltrexone effectively blocked the poststress increase in alcohol drinking. Administering naltrexone as part of the treatment for patients with both PTSD and alcoholism may help break the addictive cycle.

Margaret sought treatment from an alcoholism treatment provider after yet another extended bout with heavy drinking left her physically exhausted. During her initial evaluation, she was diagnosed with PTSD for the first time. She was referred for pharmacotherapy with naltrexone and concomitant psychotherapy using prolonged exposure, which was modified to include a focus on the functional relationship between PTSD symptoms and drinking. Prolonged exposure involves repeated, prolonged, imaginal exposure to the trauma memory or memories that arise during therapy. The patient is encouraged to relive the traumatic memory as vividly as possible, with the goal of allowing greater access to the emotional and cognitive content of the memory (which is actively avoided by individuals with PTSD) to facilitate emotional processing and integration of the experience (Foa and Rothbaum 1992). During treatment, the role of avoidance in the maintenance of PTSD was explained to Margaret, and she was encouraged to confront her traumatic past through prolonged, systematic exposure to the memories and to gradually confront the situations she had been avoiding in her daily life. During discussions of her exposure exercises, Margaret became more aware of the frequent association between her reexperiencing symptoms and her urges to drink, which through the combination of medication and the development of less avoidant coping strategies, she was generally able to resist. Over a three-month course of treatment, Margaret exhibited progressively less distress during imagined exposure, her memories for the traumatic events gradually became less disjointed, and she eventually expressed a sense of resolution regarding these events. Although Margaret appeared resistant to treatment at several points by canceling or avoiding sessions, and although she experienced two significant drinking bouts during the early stages of therapy, with the support of her therapist Margaret’s symptoms eventually diminished to the point where she could no longer be diagnosed with PTSD. As a result, she felt much more capable of combating temptations to drink, which she continued to encounter from a variety of triggers not related to PTSD. At the end of treatment, Margaret stated that for the first time in her life she felt “ free” and truly able to put her past behind her. Margaret was encouraged to maintain contact with her treatment providers for continued support to help maintain her gains and cope with setbacks.

## Summary

Uncontrollable trauma in animals and humans leads to stress-induced increases in the release of endorphins. The emotional numbing seen in rats exhibiting learned helplessness and in patients with PTSD may be related to the increased release of endorphins as a result of stress. Such increases in endorphin activity are observed in response to trauma and may also occur during exposure to trauma reminders. Afterward, a period of endorphin withdrawal may explain the physiological hyperactivity, depression, and irritability that mark patients with PTSD. This endorphin withdrawal can be reduced by alcohol use. This model has two important implications for the treatment of PTSD and alcoholism. First, therapy aimed at increasing one’s sense of mastery over traumatic events can help patients cope when exposed to trauma reminders. By reversing feelings of helplessness, one can more easily recover from PTSD and related alcohol problems. Second, the use of opioid blockers such as naltrexone may block the effects of alcohol and break the addictive cycle.
